# Improved Navigation Performance Through Memory Triggering Maps: A *Neurocartographic* Approach

**DOI:** 10.1007/s42489-024-00181-x

**Published:** 2024-12-03

**Authors:** Frank Dickmann, Julian Keil, Annika Korte, Dennis Edler, Denise O´Meara, Martin Bordewieck, Nikolai Axmacher

**Affiliations:** 1 Geography Department, Cartography, Ruhr University Bochum, Bochum, Germany; 2 Department of Neuropsychology, Ruhr-University Bochum, Bochum, Germany

**Keywords:** Cognitive maps, Neurocartography, Grid cells, Spatial memory, Navigation performance

## Abstract

When using navigation devices the "cognitive map" created in the user's mind is much more fragmented, incomplete and inaccurate, compared to the mental model of space created when reading a conventional printed map. As users become more dependent on digital devices that reduce orientation skills, there is an urgent need to develop more efficient navigation systems that promote orientation skills. This paper proposes to consider brain processes for creating more efficient maps that use a network of optimally located cardinal lines and landmarks organized to support and stabilize the neurocognitive structures in the brain that promote spatial orientation. This new approach combines neurocognitive insights with classical research on the efficiency of cartographic visualizations. Recent neuroscientific findings show that spatially tuned neurons could be linked to navigation processes. In particular, the activity of grid cells, which appear to be used to process metric information about space, can be influenced by environmental stimuli such as walls or boundaries. Grid cell activity could be used to create a new framework for map-based interfaces that primarily considers the brain structures associated with the encoding and retrieval of spatial information. The new framework proposed in this paper suggests to arrange map symbols in a specific way that the map design helps to stabilize grid cell firing in the brain and by this improve spatial orientation and navigational performance. Spatially oriented cells are active in humans not only when moving in space, but also when imagining moving through an area—such as when reading a map. It seems likely that the activity of grid cells can be stabilized simply by map symbols that are perceived when reading a map.

## Abstract (de)

Die "kognitive Karte", die im Kopf des Nutzers bei der Verwendung von Navigationsgeräten entsteht, ist viel fragmentierter, unvollständiger und ungenauer als das mentale Modell des Raums, das beim Lesen einer herkömmlichen gedruckten Karte entsteht. Angesichts der zunehmenden Abhängigkeit der Nutzer von digitalen Endgeräten, die die Orientierungsfähigkeit einschränken, besteht ein dringender Bedarf an der Entwicklung effizienterer Navigationssysteme, die die „kognitive Karte “ stärken und die Orientierungsfähigkeit fördern. Mit diesem Beitrag wird vorgeschlagen, Gehirnprozesse zu berücksichtigen, um effizientere Karten zu erstellen. Dazu könnte ein Netzwerk optimal platzierter Kardinallinien und Landmarken genutzt werden, das so organisiert ist, dass es die neurokognitiven Strukturen im Gehirn unterstützt und stabilisiert, die die räumliche Orientierung fördern. Dieser neue Ansatz verbindet neurokognitive Erkenntnisse mit der klassischen Forschung über die Effizienz kartografischer Visualisierungen. Neuere neurowissenschaftliche Erkenntnisse zeigen, dass räumlich orientierte Gehirnzellen mit Navigationsprozessen in Verbindung stehen. Insbesondere die Aktivität von Gitterzellen (grid cells), die metrische Informationen über den Raum verarbeiten, kann durch (externe) Umweltreize wie Wände oder Grenzen beeinflusst werden. Räumlich orientierte Zellen sind bei Menschen nicht nur aktiv, wenn sie sich im Raum bewegt, sondern auch, wenn er sich vorstellt, sich durch ein Gebiet zu bewegen – wie etwa beim Lesen einer Karte. Es scheint wahrscheinlich, dass die Aktivität der Gitterzellen im Gehirn allein durch kartographische Signaturen, die beim Lesen einer Karte wahrgenommen werden, stabilisiert werden kann.

## Introduction: Being Disorientated Despite Digital Navigation Aids

Digital devices such as vehicle navigation systems or map apps on mobile devices are playing an increasingly important role in spatial orientation and navigation. Many people no longer use printed maps, as mobile phones and digital navigation systems are established digital devices. Dynamic directional arrows on map displays or augmented reality elements support navigation. However, the mental representation of space (cognitive map) that is created in the user's mind when using navigation devices is significantly more fragmented, incomplete and inaccurate compared to the mental model of space created using a printed map (Dickmann 2012; Parush et al. 2007, cf. Löwen et al. 2019, Schwering et al. 2017, Anacta et al. 2017). Although digital systems reduce the temporal and cognitive effort at the moment of navigation by interacting through map displays or announcements, e.g. through auditory turn-by-turn instructions or visual cues, they do not contribute to orientation skills of their users (Ben-Elia 2021; Ahmadpoor et al. 2020). As users of navigation systems are not required to actively process spatial information to reach a destination, no survey knowledge of the space travelled through is acquired (Ben-Elia 2021; Ahmadpoor et al. 2020; Ruginski et al. 2019; Münzer et al. 2006). But how can digital systems be used and improve orientation skills at the same time?

## Efforts of Cognitive Cartography to Reduce Distortions

Increasing the efficiency of cartographic visualizations has been the focus of cartographically oriented cognition studies for decades. In the field of route based navigation research, landmarks are discussed to play a key role for orientation (Cheng et al. 2022; Credé et al. 2020; Bestgen et al. 2017; Richter and Winter 2014; Foo et al. 2005; Sorrows and Hirtle 1999). The accuracy of spatial navigation is increased using landmarks (Siegel and White 1975). A variety of topographic objects such as mountain ranges, rivers, or single buildings act as landmarks used for recognizing positions in a geographic space (Elias 2003). Landmarks are described as dominant anchor points for surrounding elements (Golledge 1991). This means that it is not necessary to memorize the absolute position of each topographic object, but only the spatial relationship (relative position) to a landmark (Keil 2021; Keil et al. 2024). Environments can be grasped in two or even three dimensions, with the help of physical landmarks that are perceived when moving through space, and with the help of landmarks that appear as symbolic representations in maps (Montello 2005; Siegel and White 1975). Interestingly, when searching for potential solutions to improve navigation processes, it is not only a single landmark in the landscape that is important for building a comprehensive understanding (survey knowledge) an environment. Rather, a network of several landmarks and the relations (e.g. distances) between them have to be considered (Bestgen et al. 2017; Schmidt and Delazari 2013; Montello 1998). The existence of a suitable network of visual landmarks is not only necessary for building linear route knowledge but, more importantly, for developing a multidimensional mental model of space (cf. Montello 2005; Fontaine 2001). However, the number of landmarks that can be used for successful navigation appears to be limited to five to seven elements due to the cognitive capacity of users (Cheng et al. 2022). Map users view landmarks as a configuration, thus structuring an environment as well as a map (Golledge 1999, 2003). Even though these illusory lines between segments do not exist on a map physically, they support building a mental representation of map-based spatial information. Recent studies point to the effect of illusory lines partitioning map space on spatial memory performance (Dickmann et al. 2017).

Accordingly, the outstanding impact of environmental cues such as landmarks on navigation and cognitive representations of space led to efforts to automatically identify appropriate spatial objects by navigation systems (Duckham et al. 2010; Elias 2003; Montello 2005). For example, automatically recorded landmarks (based on geodatabases and vehicle sensors) in an environment could be visually highlighted in navigation system displays to support orientation skills. However, defining clear rules for which environmental objects (e.g. a tower, a building or an intersection) should be selected for landmark visualization in a navigation system is a pressing problem in cartographic and spatial cognition research that has not yet been solved (Nuhn and Timpf 2019; Quesnot and Roche 2015; Montello 2009; Elias 2003; Raubal and Winter 2002). Humans intuitively assign personal experiences or meanings to landmarks (Sorrows and Hirtle 1999). Their emotional states can also influence navigation performance (Aly et al. 2024; Lanini-Maggi et al. 2023; Gardony et al. 2011). In addition to these individual dimensions of experience, other factors contribute to categorizing spatial objects as a landmark. For example, landmark use is known to depend on context (Richter and Winter 2014) and the specific task at hand (Montello et al. 1994). Furthermore, recent studies show that for route learning using 2D maps (orthogonal view), only local landmarks, i.e., cartographic cues close to the route to be learned, are utilized (Keil et al. 2018, 2020a, b), whereas global landmarks are more important in 3D navigation and for gaining survey knowledge (Credé et al. 2020). Krukar et al. (2020) were able to show that local and global landmarks lead to different sketch maps and thus to different cognitive representations of space.

Obviously, the search for a successful landmark prediction model requires knowledge about cognitive processes during navigation. Numerous empirical studies provided consistent evidence that adding space-referencing elements to topographic maps facilitates distance estimations and enhances memory for object positions (Edler et al. 2014, 2015, 2019; Dickmann et al. 2015, 2013). However, underlying cognitive mechanisms as to why this works remain unclear. To gain a better understanding of the processes involved, it is important to consider the associations between map reading and the related brain functions.

## Map Based Concepts Addressing Brain Processes

In recent decades, spatial cognition research brought forth important findings that appear promising for developing efficiency-enhancing map designs. Maguire et al. (1998) demonstrated different functions of the right and left brain hemispheres for navigation. Results from cognitive science also show that categorical assignments, such as the information that a building is located to the south or north of a street or that an object belongs to a superordinate (coarser) spatial unit, are primarily processed in the left hemisphere of the brain. In contrast, precise (metric) representations, such as the exact distance of a building to a street, appear to be processed in the right hemisphere (cf. van Asselen et al. 2008; Kessels et al. 2002; Kosslyn et al. 1989). It appears that objects are not only perceived and cognitively processed with regard to their position in relation to other objects on the map, but also—on a further level—according to coarser spatial units (categorical). It can be assumed that superordinate spatial units are processed separately from the detailed topographical information supporting the recall of correct object positions or distances (Dickmann et al. 2019). The distribution of spatial memory processes (object-location memory) in different areas of the frontal, medial temporal and parietal cortex (van Asselen et al. 2008; Roseman et al. 2024) could explain why, for example, the use of additional grid geometries integrated into maps leads to an increase in memory performance (Dickmann et al. 2015, 2013; Edler et al. 2014, 2015).

The idea of also using spatial representations that are less precise but much easier to recall appears to make sense from an evolutionary perspective if the combination of categorical and geometric knowledge is associated with an overall reduction in distortions and is, hence, adaptive (Waller and Nadel 2013). However, this does not completely prevent the emergence of distortion, as assigning locations to broader, higher-level (categorical) spatial units inevitably results in less accurate memory recall of specific positions. During recall, there tends to be a shift towards the category center, which is referred to as "central tendency bias" (Huttenlocher et al. 1991; Duffy et al. 2010; cf. Korte et al. 2023). Nevertheless, the average error is minimized, and the recall of information is decisively supported (Richter and Winter 2014; Stevens and Coupe 1978).

**Fig. 1:**
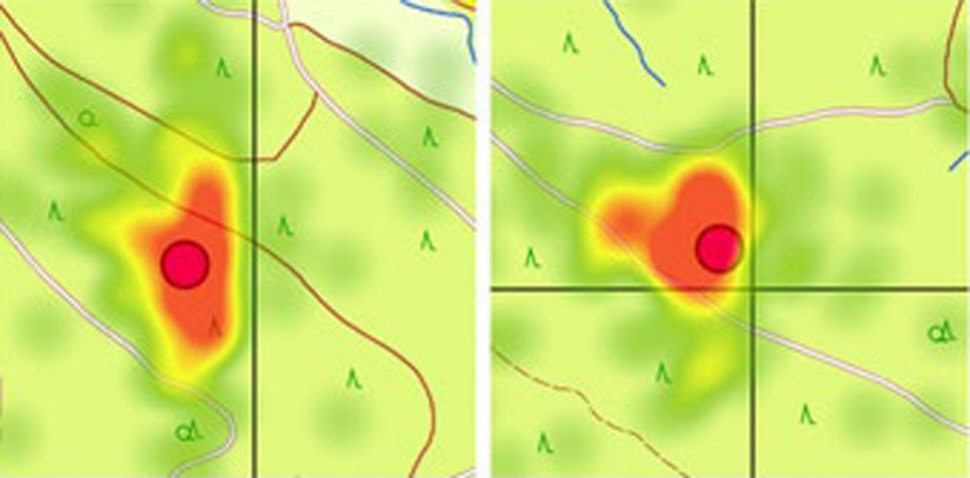
In a topographic map, grid geometry (black lines) apparently influences eye movements of a participant (fixations represented as green, yellow and red dots as the number increases) that focus on an object (red circle) during an object location encoding (Dickmann et al. 2015)

For the cognitive processing of cartographic information, this means that perceiving precise (metric) positions of map elements activates the right hemisphere of the brain, while perceiving categorical information (e.g., assignment to a region or grid field) activates neuronal networks in the left hemisphere of the brain (cf. Kosslyn et al. 1989). Data from eye-tracking studies seem to support this idea (Kuchinke et al. 2016; Dickmann et al. 2015). Analyses of eye movements during the encoding phase of a memory task show that encoding of a map, which is partitioned into coarse sub-areas (categories) by graphic grid lines, is influenced by the boundary lines (Fig. 1). This could indicate the fundamentally separate processing of detailed and coarser spatial information in the brain. The perception of aggregated spatial information, such as areas surrounded by boundary lines in a map, causes a systematic distraction of gaze behavior. This possibly mirrors the categorical processing of spatial information in the right brain hemisphere, which merely encodes assignments of objects to superordinate spatial units instead of precise (metric) object positions.

The arrangement of legends (textual information) in maps also addresses the different functions of the left and right hemispheres of the human brain (Edler et al. 2020). In around 95% of all right-handers (and 60% of all left-handers), language and text processing takes place predominantly in the left hemisphere of the brain (Gazzaniga and Smylie 1984; Springer and Deutsch 1997; Knecht et al. 2000). This functional asymmetry seems to offer the possibility of addressing specific brain activities to increase memory performance. Edler et al. (2020) showed that the processing of text information (legend information) is significantly faster if it is transferred as directly as possible to the left hemisphere of the brain during the perception process (cf. Willemin et. al. 2016). For a faster and more efficient processing of textual map information, it would be more advantageous to generally place legend or text information in the right half of the visual field.

Auditory and visual verbal stimuli/information (heard and written/read words) are/is processed in completely different areas of the brain (cf. McEachren 2004). Multimedia maps therefore have the immediate effect of activating different regions of the brain when auditory as well as visual map elements are used. In addition, the identity and location of spatial objects are also stored separately from each other (Darling et al. 2006; Pertzov et al. 2012; Walter et al. 2003). When retrieved as one (intergrated) representation, both object properties must be brought together by the object-location association process (binding) (Lehnert and Zimmer 2008). As a third component, binding is crucial for successful recall and depends on the posterior parietal cortex (Kesner and Creem-Regehr 2013). Spatial objects are therefore not learned as a whole (i.e. object position and object identity). From a cartographic point of view, however, the combination of both object properties is an indispensable condition for the successful transfer of spatial information (Dickmann 2018, 126). The sole retrieval of an object location without information about the object identity is of little use for any spatial task. Lammert-Siepmann et al. (2017, 2020) demonstrated that a reinforcement of the semantic information (identity) of place names in a map, which is evoked by simultaneous visual (text) and spoken information, results in a stronger binding of identity information and position information (i.e. thus possibly an increase of brain activity in the posterior parietal cortex). These few examples point to the potential of considering brain processes to create more efficient maps.

## Neuroscientific Approaches

In order to gain further insights into the interaction of map elements and cognitive processing, methods from brain research are increasingly being applied (Fabrikant 2023; Lobben et al. 2019). In particular, the recent results of fMRI studies on neuronal networks of spatially coordinated cells (Peer and Epstein 2021; Huffman and Ekstrom 2019; Maidenbaum et al. 2018) underline the idea of how cartographic information is processed in different brain regions. Based in part on findings that (punctual) landmarks and boundaries are processed in different brain regions (Doeller et al. 2008, Doeller and Burgess 2008), a first taxonomy of external cues for navigation has already been proposed (Chan et al. 2012).

Innovative concepts have been proposed that start explicitly from the individual user to meet the challenge of improving navigation abilities (Fabrikant 2023, 2022; cf. Reichenbacher 2001, 2023). The aim is to develop neuroadaptive systems that take into account human-centered navigation conditions such as the spatial abilities, the changing decision-making contexts and the neurocognitive/psychological resources of users during navigation (using mobile geographic information displays). Neuroadaptive technology could monitor, for example, the user's level of mental workload and react on the fly to adapt interface elements (on mobile geographic information displays) that may influence further information processing (Fairclough 2022). Maps then could be adapted, e.g., by displaying additional information or suppressing superfluous information to increase learning depending on workload or emotional state. Initial studies on the methodological principles show how electroencephalographic (EEG) data, eye-tracking data and recordings of electrodermal activity during map-assisted navigation can successfully serve as real-time indicators of physiological and neurocognitive states (Fabrikant 2023; Cheng et al. 2022; Keil 2021). In the future, technical miniaturization could lead to a practical implementation of such neuroadaptive systems.

A promising approach to increasing the efficiency of maps for navigation could involve not only individual and context-related dimensions of navigation but also incorporating map features that support the functioning of spatial brain structures. Thus, regardless of the individual situation in which a map is used, map features could contribute to the activation of brain regions with important functions in the processing of spatial information. It can be assumed that neuroscientific findings on spatially tuned cells in the brain could lead to a further step towards understanding how maps improve navigation performance. They could expand map theory and have practical implications for the optimization of map designs (McEachren 2004). The maps could then be designed in such a way that their main graphical structures are optimally adapted to the activity profiles of spatially tuned cell populations in the brain. This could be achieved, for example, by using landmarks (positions) in virtual reality (VR) environments and in maps that are tailored to the properties of spatially responsive cells. However, empirical findings on the influence of cartographic cues (map elements) on neural processing and a systematic understanding of how such issues could contribute to improvement spatial abilities are still lacking.

### Spatially Tuned Cells in the Brain

Recent advances in the neuroscience of navigation and spatial memory allow for assigning much more precise functions to specific areas within the brain and its functional cell types. It is known that spatial information is processed in differently specialized brain systems (Giocomo et al. 2011, O’Keefe and Dostrovsky 1971). Neuroscientific studies on the processing of spatial information in the brain of animals (Hafting et al. 2005; Hardcastle et al. 2015) show the role of spatially tuned cells, such as place cells, grid cells, border cells etc.—for navigation and spatial memory (Bellmund et al. 2016; Moser et al. 2015; Kunz et al. 2019). Evidence for grid cells in humans has been provided by Jacobs et al. (2013). Specific activity patterns have been found in the human brain that preferentially respond to specific locations or movement directions (Seeber et al. 2024; Julian et al. 2018; Nau et al. 2018; Doeller et al. 2010). In the search for an improved navigation theory, such evident interactions between cell network and navigation processes are crucial. Spatially tuned cells in the mammalian hippocampal and parahippocampal cortices are important for the cognitive mapping of the spatial environment (Derdikman and Moser 2014). Their characteristic firing patterns lead to a microstructure of a spatial map in the hippocampus (Hafting et al. 2005; Solstad et al. 2008), enabling individuals to self-locate and navigate in different environments.

These findings on the neuronal structures of the human cognitive map have the potential to be used in cartographic research for the further development and redesign of maps. Linking the mechanisms of cartographic information transfer with spatial memory processes can provide a new framework for map theory if it is possible to take neuronal structures into account in the design and arrangement of map symbols. These latest neuroscientific findings could be used to strengthen the functional interaction between external cartographic (spatial) information and the neuronal networks in the brain that are responsible for spatial navigation and orientation. Maps could then be designed so that their main graphical structures are optimally adapted to the activity profile of populations of spatially tuned cells.

This means that the neuronal networks responsible for spatial memory have to be influenced or triggered by external cartographic cues. The specific properties of different neuronal networks and their connections provide crucial information. First in the rat brain, but recently also in the human brains, activity has been found that preferentially responds to a specific location or movement direction (Julian et al. 2018; Nau et al. 2018; see Hafting et al. 2005). To date, different classes of spatially tuned cells have been found, including (among others):

- place cells (O’Keefe and Dostrovsky 1971),
- head direction cells (Taube et al. 1990),
- grid cells (Hafting et al. 2005),
- border cells / boundary vector cells (Solstad et al. 2008),
- speed cells (Kropff et al. 2015),
- object-vector cells (Høydal et al. 2019),
- vector trace cells (Poulter et al. 2021).

**Fig. 2:**
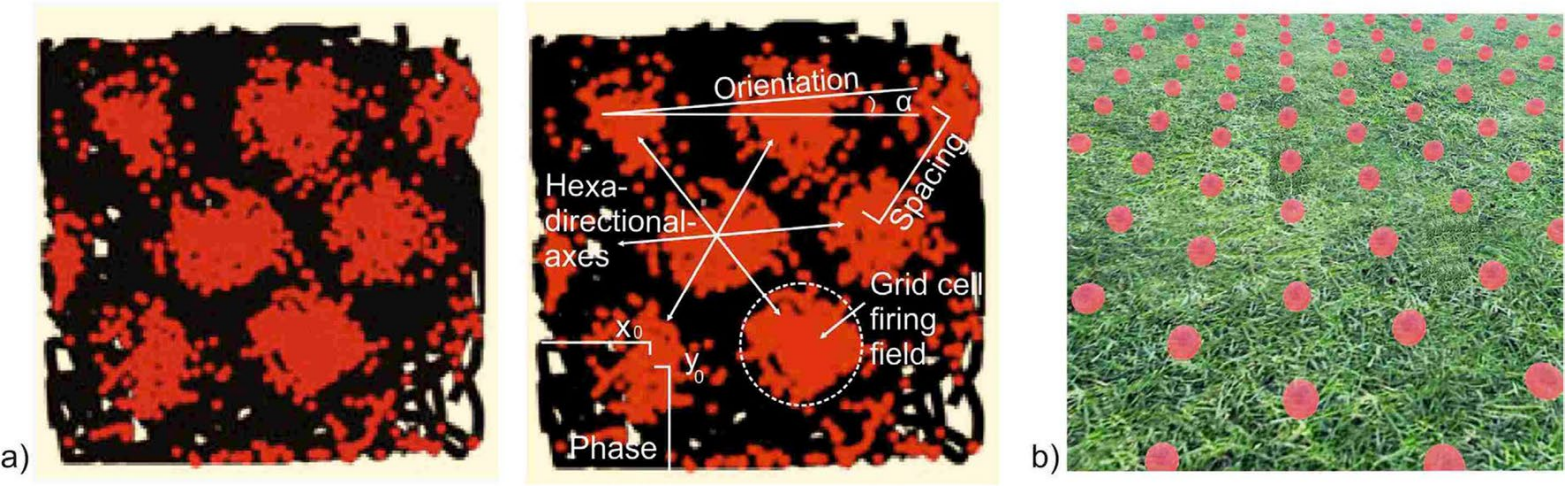
**a **Geometric pattern of grid cell firing fields (here: grid cell example of a rat); red dots mark the firing positions of a grid cell of a rat moving around in a box; the rat tracks shown in black (since the rat ran through almost the entire area (cage) during the electrophysiological measurement with revisited locations where grid cells fire, the area appears almost completely black); grid cell firing fields exhibiting a characteristic sixfold rotational symmetry (Bonnevie et al. 2013; with permission, license number 5730791287020); grid cell properties: spacing, phase ( *x*_0_,*y*_0_), and orientation (*α*) (according to Derdikman and Moser 2014) (Bonnevie et al. 2013; Moser et al. 2014); **b** assumed sketch of grid cell firing fields (activated in the brain) when moving through a grassy environment

Place cells discovered several decades ago in rats fire at unique spatial locations in the environment and thus provide information about one’s current position (O’Keefe and Dostrovsky 1971). These neurons in the rat hippocampus fire only when the animal visits or revisits a certain spatial location (Giocomo et al. 2011). This was the first indication of the existence of an “inner map” at the single-neuron level. However, it initially remained unclear what triggers the activity of place cells in the brain. In the following decades more spatially tuned cells such as head direction cells encoding an animal´s direction, and border cells encoding borders within an environment, have been found. These cells respond specifically to external cues such as walls or other borders in the environment (Solstad et al. 2008; Taube et al. 1990). Most interesting for the explanation of navigation behavior is the discovery of grid cells (Hafting et al. 2005) (Fig. 2). Their characteristic firing patterns lead to a microstructure of a "spatial (inner) map" in the entorhinal cortex (Hafting et al. 2005; Solstad et al. 2008), probably providing the spatial metric for navigation (Bush et al. 2015). Animal experiments have shown that when an animal starts to move through an environment, the activity of these brain cells reflects a geometric grid pattern with a pronounced six-fold rotational symmetry. The characteristics of grid cells exhibit repeated firing fields that cover the vertices of equilateral triangles, effectively tiling the environment (Bush et al. 2015) (Fig. 2). The existence of such an inner metric system could possibly play a central role in route planning and map reading.

### Grid Cells

**Fig. 3:**
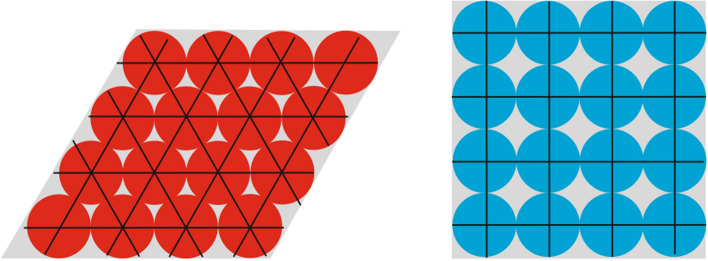
Potential advantages of six-fold over four-fold grid cell firing fields. The hexagonal pattern enables a higher directional resolution as six directions (3 axes) are available instead of only four (2 axes). With the hexagonal arrangement, a higher, more efficient packing density (few gaps) can be achieved to cover the surrounding area

The geometric firing pattern of grid cells is considered part of an internal navigation mechanism (inner map) that serves as a basic function for dividing a homogeneous area (open field) into geometric units (Bush et al. 2015). The regular distance between the firing fields provides an accurate measure of the distance traveled in any given environment (Fig. 2). Their systematically repeating firing fields can be deemed optimally suited to support distance estimation and path integration, as they are considered to possibly provide the computational basis for representing navigation processes in the brain (Burak and Fiete 2009). The hexagonal arrangement has obvious advantages for the processing of navigation processes in the brain (e.g. Stemmler et al. 2015) (Fig. 3). Their most striking feature is that these cells maintain their basic hexagonal firing pattern over a long time period, even if the directional movement or the environment changes (Rowland et al. 2016). As part of an inner map the hexagonal firing fields, thus, likely form the basis for an allocentric representation of space. Metric information can be used to explore new connections between objects, e.g. short cuts (Werner et al. 2000). Recent studies have also shown that this grid-like structure not only appears in navigation tasks, but also in the representation of more abstract information such as feature and concept spaces (Nitsch et al. 2024; Chen et al. 2024; Whittington et al. 2022; Bellmund et al. 2018).

In terms of spatial orientation, studies have shown that the six-fold rotational symmetry of regular grid cell firing fields is mainly developed in open field environments (He and Brown 2019), i.e. when no further spatial cues are available and navigation cannot be based on alternative navigational strategies (Bierbrauer et al. 2020). Grid cells can differ from each other by orientation, spacing (distance between grid fields) and phase (position of grid field center along hexadirectional axes) (Derdikman and Moser 2014; Hafting et al. 2005). However, not just one grid cell, but ensembles of local cells (grid cell arrays) with a common grid spacing and common orientation represent the surface of an environment. Slightly shifted phases of neighboring cells of an ensemble can completely cover an environment with vertices of equilateral triangles (Hafting et al. 2005). In addition, grid cells are organized in modules (Rowland and Moser 2014). In contrast to the grid cell arrays, modules also have a uniform orientation and scaling, but differ from each other by different spacing, and they are spatially separated from each other in the entorhinal cortex (of an animal) (Brun et al. 2008; Hafting et al. 2005). The differences in spacing range from a few decimeters to several meters, whereby individual levels can be distinguished with a scaling factor of approx. 1.4 (Moser and Moser 2016). It is therefore assumed that the modules can be used to represent spatial expansions at different resolutions (distances) (Derdikman and Moser 2014). Most importantly, this organization in modules of grid cells with different scaling is the computational basis for unambiguously determining an organism’s exact local position in the environment it traverses (Dong and Fiete 2024).

Although neuroscientific studies are mainly based on single-cell recordings in animal experiments, similar findings can also be obtained in humans using intracranial EEG recordings in presurgical epilepsy patients (Chen et al. 2018; Maidenbaum et al. 2018) and functional magnetic resonance imaging (fMRI) in healthy participants (e.g. Bierbrauer et al. 2020; Kunz et al. 2015). FMRI is a type of neuroimaging technique that allows conclusions to be drawn about neuronal activity based on changes in the oxygenation of blood flow in the brain. Thus, in humans, the activity of grid cells can be measured indirectly as "grid cell-like representations", GCLRs (Doeller et al. 2010). These GCLRs are considered functionally relevant for spatial memory and spatial navigation (Bierbrauer et al. 2020; Kunz et al. 2015; Doeller et al. 2010). Doeller et al. (2010) found significantly higher grid cell-like activity in the human entorhinal cortex when participants virtually moved along six hexadirectionally symmetric directions (Fig. 2). These preferred directions, reflecting the hexagonal grid cell firing fields, are determined by environmental boundaries or by cardinal axes (Nau et al. 2018; Navarro Schroeder et al. 2020; Bellmund et al. 2016).

### The Role of External Cues for Grid Cell Based Navigation

It is assumed that grid cell activity arises primarily from intrinsic local network dynamics and input from other spatial cell types but also from direct extraction of features from sensory input (Burgess and O’Keefe 2011; O’Keefe and Nadel 1978; Widloski and Fiete 2014). The grid orientation (of a grid cell array) is substantially affected by boundaries of a spatial environment, e.g. walls in test boxes of animal experiments (Krupic et al. 2015). Recent studies also found “shearing” effects inducing grid rotations (Stensola et al. 2015) and distortions of grid fields when boundaries (walls) were displaced (Barry et al. 2012). These findings show that grid cells appear to be anchored to an external reference frame such as environmental boundaries. Such external cues correct the accumulating errors that occur when the activity of the grid cells depends solely on internal brain network dynamics (Hardcastle et al. 2015; Julian et al. 2018; Stensola et al. 2015). This underlines the important role that sensory cues have for building spatial representations. Employing intracranial electroencephalography (iEEG) Chen et al. (2018) found evidence for stronger grid cell activity (grid cell-like representations in humans) in parts of the environment that were closer to the environmental boundary. This effect is possibly also related to the stabilization of grid cell firing by error-correcting input (e.g. from border cells) after encounters with environmental boundaries (Hardcastle et al. 2015).

**Fig. 4:**
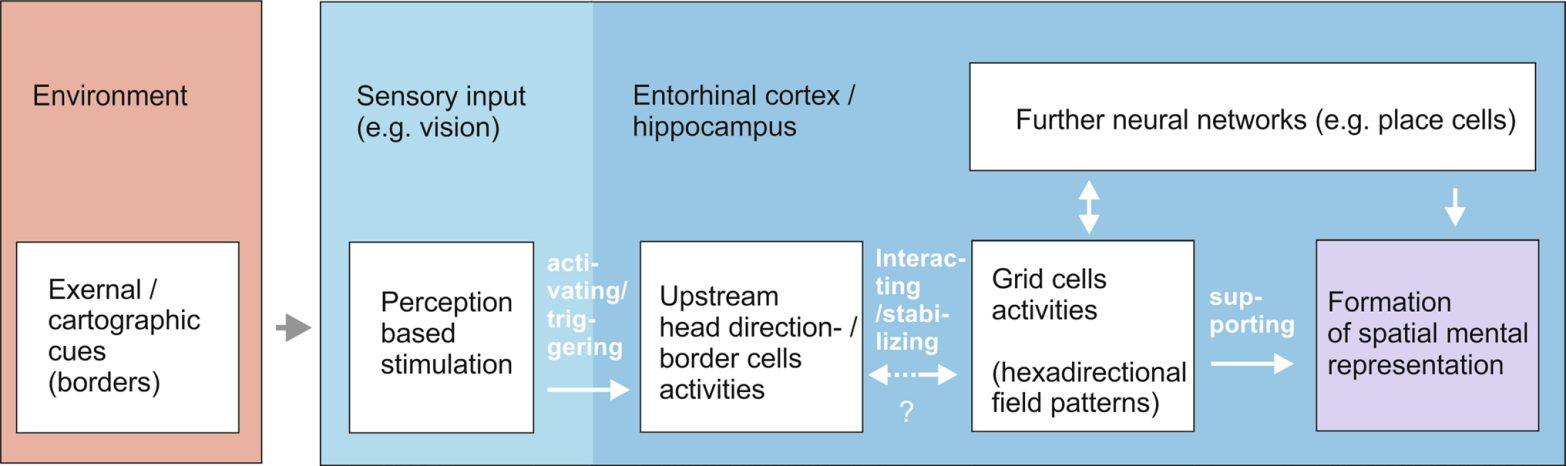
Assumed influence of cartographic cues on grid cell activity and the formation of a spatial mental representation (simplified according to Winter and Taube 2014; extended)

In humans, the activity of grid cells is also influenced by the presence of environmental markers (punctual markers) and boundaries (Bierbrauer et al. 2020). Navarro Schroeder et al. (2020) found that the path integration performance depends on the relative orientation of polarized distal cues (a cardinal axis) of an environment to the traveled directions. The orientation of the six-fold rotational symmetry of grid fields seems to have an impact on the way an environment is perceived. Apparently, cardinal axes created by environmental cues, e.g. by a mountain range or the north–south axis in an environment, may have a stabilizing effect on the orientation of grid cell firing fields. Cells upstream of the grid cells, such as head direction cells and boundary cells, can help stabilize the firing fields of the grid cells, as they specifically respond to external cues (Solstad et al. 2008; Taube et al. 1990). Environmental cues are perceived by different sensory modalities such as vision, olfaction, or tactile sensations (Richter and Winter 2014; Winter and Taube 2014; Solstad et al. 2008). Recent studies show that even social cues have impact on spatial cells firing (Xu et al. 2022). These functional cell types are important for creating external reference frames for spatial information. The six-fold rotational symmetry of a regular grid cell aligns with a superior cardinal axis, thus anchoring the hexadirectional pattern (Navarro Schroeder et al. 2020). After sensory encounters with e.g. environmental boundaries, upstream border cells may contribute to the firing of grid cells through external error-correcting inputs (Hardcastle et al. 2015) (Fig. 4). Stabilized grid cell activity could therefore be part of a mechanism to encode locations in (visual) space in such a way that lasting spatial representations can be created.

It appears that mapping an environment depends on sampling spatial information near axes or nodes of the hexagonal grid, which results in an increase in firing activity (Killian and Buffalo 2018). The geometric pattern of the grid cell firing fields points to the existence of an allocentric memory structure that can be used for orientation independently of the (egocentric) eye and head movements of the perceptual process (Julian et al. 2018). Such allocentric processes store spatial information in the long term as spatial mental representation (“inner map”) and could be used for real word navigation or route planning (Waller and Nadel 2013).

### Grid Cell Activity During Visual Exploration and Map Reading

Of particular interest to (cognitive) cartography are the findings that grid cell firing patterns appear to be elicited not only as a result of physical movement through an environment, but also but also on location-based visual exploration (Julian et al. 2018; Kuhrt et al. 2021; Killian and Buffalo 2018; Nau et al. 2018). These studies show that “spatially periodic neuronal activity arises remotely, without physical visits to any locations within the environment” (Killian and Buffalo 2018). A first approach to investigate grid cell activity while solely viewing of (two-dimensional) images was pursued by Killian et al. (2012). They examined spatial representations in the entorhinal cortex (EC) of head-fixed monkeys performing a two-dimensional (map-like) visual exploration task. Interestingly, the results provided evidence that “EC neurons encode space during visual exploration, even without locomotion “ (Killian et al. 2012). Specifically, they could demonstrate that gaze movements are associated with grid cell firing while the animals visually explored images. Similar results were found for grid cell-like representations using fMRI and eye-tracking in humans (Julian et al. 2018). This latter study revealed a sixfold modulation of the fMRI signal (0°, 60°, 120° …) as a function of gaze-movement direction when participants were solving a search task on a display (see also Nau et al. (2018) for a similar result). Gaze movements aligned with the three (bidirectional) hexadirectional axes show greater entorhinal cortex activity (area where grid cells are found). In particular, fMRI activity was reported to be greater for gaze movements that were aligned compared to those that were not (Julian et al. 2018; Nau et al. 2018).

Importantly, the grid orientation rotated in concert with the rotation of the search display, again showing alignment of grid axes with visual borders. Finally, Bellmund et al. (2016) and Horner et al. (2016) provided evidence for grid cell-like representations when participants only imagined self-motion along a hexadirectional axis. This means imagining or remembering spatial scenes elicits similar grid cell activity as experiencing the real world through physical movement or eye movements. It seems likely that grid cells also fire in a predictable manner during a map-based (2D) exploration of an environment, especially when imagining moving through the depicted environment.

## How can Neuroscientific Findings be Used to Support Navigation and Map Reading?

It can be assumed that building a mental representation of an environment could be significantly improved, if the design of spatial visualizations is adapted to the functioning of the corresponding spatially oriented cells. For example, Maidenbaum et al. (2018) and Killian and Buffalo (2018) found evidence that fMRI signals in the human brain, which are considered to specifically represent grid cell firing activity (Doeller et al. 2010), closely interact with navigation performance. Since both navigation in the immediate environment and navigation with maps naturally use external (mostly visual) cues, the firing of grid cells could possibly be influenced or even controlled by grid cell-aligned cardinal axes or boundaries. Maps usually consist of a two-dimensional configuration of visual signs representing places (cities, lakes, forests) or spatial frames (roads, borders, rivers), some of which may be suitable to act as external cues modulating grid cell firing. As this concept directly addresses spatially oriented cells in the brain, it can also be assumed that the performance-enhancing effects do not require any additional learning effort.

The neuroscientific findings do not only provide explanations of the neuronal basis of navigation processes. Evident interactions between cell networks, external cues and navigation processes also point to mechanisms to improve navigation performance. These interactions, which for the first time enable a direct link between neuronal processes in the brain and the perception of spatial structures, form the basis for the derivation of a new cartographic theory that could significantly expand our understanding of how external maps function and can be used more efficiently.

Empirical studies using electroencephalography (EEG) or functional magnetic resonance imaging (fMRI) should shed more light on the neural bases of human navigation capabilities to clarify how they can be influenced by cartographic designs. In order to test the effectiveness of the new map theory, such neuroscientific analysis techniques must be combined with methods of cognitive cartography, such as eye movement registration and the measurement of spatial memory performance, when map symbols (cardinal axes, landmarks) are used that could influence grid cell activity.

*The example of cardinal axes*: Neuroscientific studies show that in order to stabilize grid cell activity, there must be an external cue (framework) such as a predominant wall or a boundary in an environment (Julian et al. 2018; Chen et al. 2018; Hardcastle et al. 2015; Stensola et al. 2015). Therefore, cardinal axes of an environment seem to be promising to support/trigger the firing pattern of spatial oriented cells neuronal activities externally. Here, polarizing cues (Hafting et al. 2005) or axes (Schroeder Navarro et al. 2020; Nitsch et al. 2024; cf. Sturz et al. 2011 and McNamara et al. 2003) for the role of cardinal axes for spatial learning) in an environment play a crucial role by serving as anchors to which the grid cell firing fields can align. This neural mechanism may explain why the use of common reference directions is important for navigation in larger environments (cf. Werner and Schmidt 1999). In a large-scale environment, a cardinal axis such as a mountain range, a river or a coastline could represent an external reference frame. However, even represented in two-dimensional maps (small-scale), it seems likely that external cues could support grid cell firing. Previous spatial cognition studies on landmark use in maps already revealed the impact, which cardinal axes specifically have on eye movements (Keil et al. 2020b, 2024). Their findings show that landmark pictograms close to (even imaginary) cardinal axes of to-be-learned objects within a map frame were fixated more often. Apparently, cardinal axes are processed in the preliminary stage of spatial perception (see above).

**Fig. 5:**
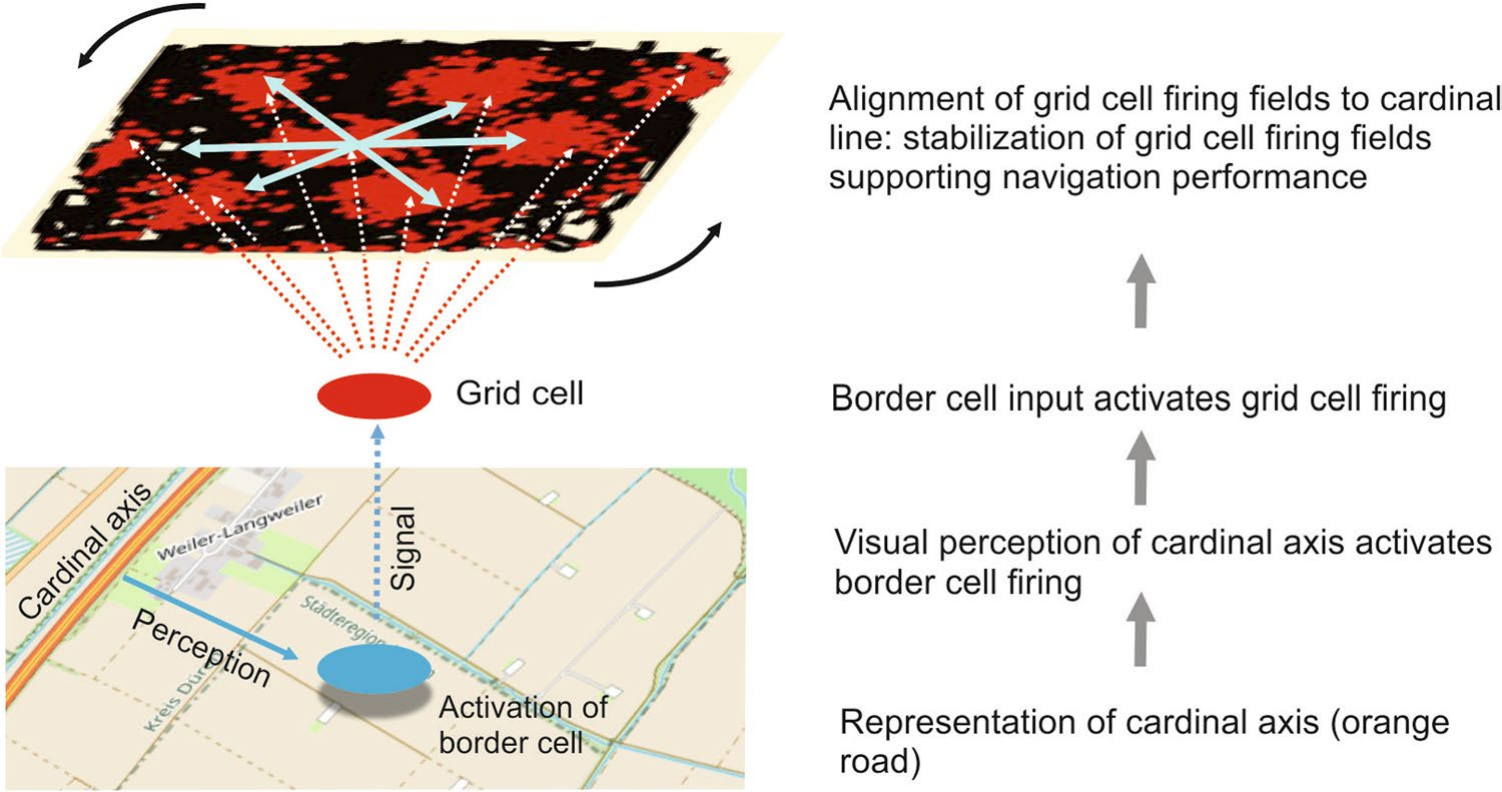
Conceptual approach to trigger memory performance: using map elements such as represented cardinal axes to stabilize grid cell firing in the brain (cell firing image, Bonnevie et al. 2013; map data © OpenStreetMap contributors, licensed under the Open Database License (ODbL). Retrieved from www.openstreetmap.org)

In a map, a predominant boundary or a cardinal axis can be considered as a subclass of landmarks that are characterized by their geometric (linear) shape but are not associated with a single environmental point (Cheung et al. 2012). Based on neuroscientific findings on the properties of grid cells, the perception of a cardinal axis (in an environment or on a map) has an effect on the firing fields of the grid cells (of a single grid cell). Presumably, border cells are involved in the transmission of signals to the grid cells, which then become (more) active. The firing pattern aligns with the cardinal axis and stabilizes (Fig. 5).

**Fig. 6:**
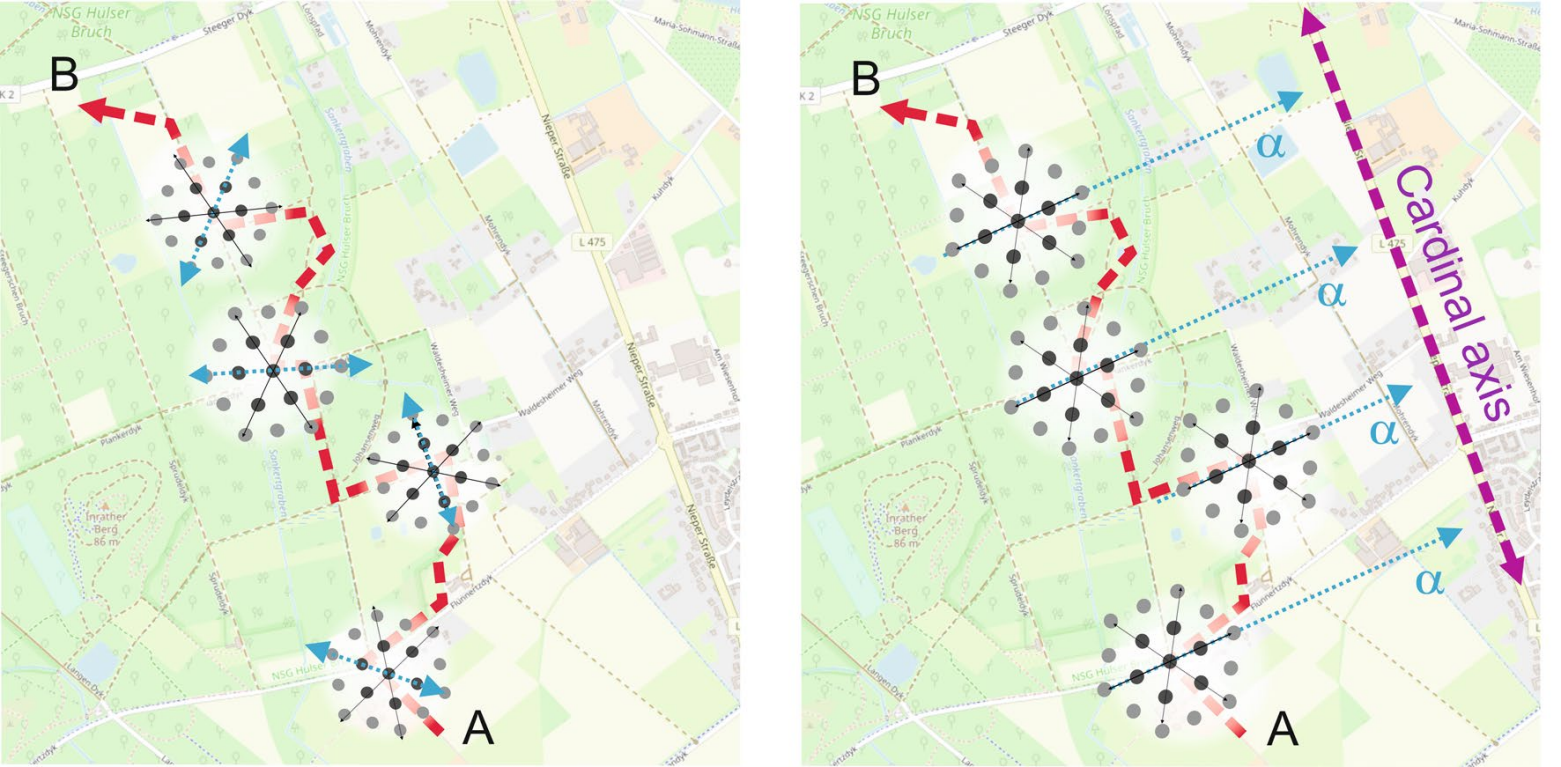
Visualizing a cardinal axis of the environment as a superordinated reference presumably maintains the alignment of grid cell firing fields (cf. Navarro Schroeder et al. 2020; Hafting et al. 2005), thus stabilizing the grid cell firing fields and supporting navigation (building spatial mental representation; map data © OpenStreetMap contributors, licensed under the Open Database License (ODbL). Retrieved from www.openstreetmap.org)

The stabilization of grid cell firing fields based on cardinal axes within an environment could probably prevent that spatial environments in the entorhinal cortex and hippocampus have to be represented as a mosaic of fragmented sub-maps of grid cell firing (Fig. 6). This would be expected when moving along a route characterized by changing environments, which would necessitate constant realignment of the grid firing fields (Derdikman et al. 2009). Stabilizing the six-fold rotational symmetry of grid cell firing patterns through a cardinal axis may allow individuals to better locate themselves and to navigate to a goal location. Since axes create an external reference frame to which the firing fields of the grid cells are aligned (Hardcastle et al. 2015; Stensola et al. 2015), a predominant axis such as a large road in the visual space of an environment or represented in a map could possibly support the firing of the grid cells.

With the help of augmented reality objects, corresponding cardinal axes could be projected into the field of vision (windshield of a car or using AR glasses). This could also be achieved in two-dimensional maps, for example, by graphical highlighting. It can be assumed that external cues such as cardinal axes influence perception and memory, regardless of whether they are perceived directly during navigation or must be transformed from an allocentric (map-based) to an egocentric spatial frame of reference. This transformation is an elementary prerequisite for capturing spatial information from two-dimensional maps. Therefore, the expected effects (of cardinal axes on spatial memory and navigation performance) are likely to be less pronounced with maps than when navigating through real or VR based 3D spaces.

Interestingly, the results of anthropological research on spatial navigation also support the idea of enhancing maps using cardinal axes. Indigenous peoples have been found to have navigational strategies based mainly on the use of cardinal axes of the environment (Haviland 1998; Levinson 1997). Urban planning theory, such as K. Lynch's notion of the special importance of edges for better navigation through cities (Lynch 1968), also provides interesting clues.

## Outlook

In the last two decades, neuroscientific findings on the interplay between environmental influences and neuronal networks in the human brain have made great progress, offering new perspectives for map design. Possibly, the construction of a mental spatial representation could be improved if the design of spatial visualizations was specifically adapted to the neuronal mechanisms that support grid cell firing. The idea of creating cartographic designs based on neuroscientific findings still requires thorough empirical testing. Up to now, it has not yet been clarified to what extent and with which characteristic features visual cues can be used to influence the activation of spatially tuned cells and navigation performance. It is hardly possible to control the appearance of external (physical) cues in the real world, when travelling through an environment. However, there is the option of exercising control over a spatial scene by using cartographic models. Navigation performance in a VR environment (3D) or even based on map reading (2D) can be expected to improve when users are presented with visual elements that match with the geometric patterns of the grid cells that fire the fields.

Media such as images, maps (navigation system displays), virtual reality (VR), or augmented reality (AR) could be used to enhance the formation of mental representations of an environment. Highlighting specific map elements and inserting additional graphic structures may align grid cell firing in a controlled manner. Virtual or augmented reality elements could also display external cues (cardinal axes), even if they are not in the viewer's immediate field of vision, e.g. a river that is obscured by a forest or a row of buildings in the surroundings.

**Fig. 7:**
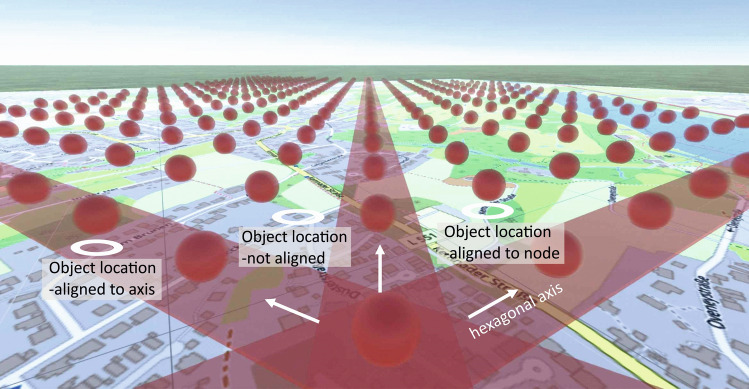
Moving along a hexagonal axis in a 3D environment (or even imagined moving) when observing navigation system displays or reading maps possibly supports spatial memory performance; object locations on a map (white circles) aligned to hexadirectional axes and nodes of grid cell firing fields (here represented by red bands and spheres) are probably better learned. (Map data © OpenStreetMap contributors, licensed under the Open Database License (ODbL). Retrieved from www.openstreetmap.org)

The consideration of cardinal axes, which serve as anchors for the stabilization of grid cell firing, could be a first step. In addition, other properties of grid cells could be focused on, such as the characteristic hexagonal configuration of grid cell firing fields. Presumably, the hexadirectional axes or the spacing of the grid cell firing pattern could also be used to enhance navigation performance (Fig. 7). When moving through a (VR) environment, viewing dynamic navigation displays, or simply imagining traversing an environment (map), spatial objects could affect navigation performance depending on whether or not they are aligned with the hexadirectional axes of the inner map from the observer's perspective (cf. Maidenbaum et al. 2018; Doeller et al. 2010). It can be argued that environmental objects that spatially coincide with these inner triangular nodes are likely to be processed in the brain more efficiently than objects between two nodes.

If the design of spatial visualizations is adapted to the computational principles of the relevant neuronal structures, the activation of spatially tuned cells could probably be accelerated, supporting, for example, distance estimations between two cities represented in a map. It can be assumed that all cartographic media, e.g. screen-based 3D visualizations or virtual or augmented realities (VR/AR) (Dickmann et al. 2021; Keil et al. 2020c) could benefit from such “neurocartographic” designs. Virtual environments even allow users to “locomote” artificially through a 3D environment (Keil et al. 2021), thus simulating movement that is important for stabilizing grid cell firing. Maps are widely used to indirectly explore an unknown area by solely cognitively processing viewed represented landscapes (imagining to be inside that area or move through it). Both, the artificial locomotion through a virtual environment (simulating to some extent proprioceptive processes), and the perception based on mere visual exploration of a map would likely recruit the activity of grid cells. The implementation of elaborated cartographic cues may then improve navigation performance in real and virtual environments.
